# Anti-Ad26 humoral immunity does not compromise SARS-COV-2 neutralizing antibody responses following Gam-COVID-Vac booster vaccination

**DOI:** 10.1038/s41541-022-00566-x

**Published:** 2022-11-15

**Authors:** Maria G. Byazrova, Ekaterina A. Astakhova, Aygul R. Minnegalieva, Maria M. Sukhova, Artem A. Mikhailov, Alexey G. Prilipov, Andrey A. Gorchakov, Alexander V. Filatov

**Affiliations:** 1 Laboratory of Immunochemistry, National Research Center Institute of Immunology, Federal Medical Biological Agency of Russia, 115522 Moscow, Russia; 2 Department of Immunology, Faculty of Biology, Lomonosov Moscow State University, 119234 Moscow, Russia; 3 Department of Immunology, Institute of Medicine, Рeoples Friendship University of Russia (RUDN University) of Ministry of Science and Higher Education of Russia, 117198 Moscow, Russia; 4 Institute of Science and Technology Austria (IST Austria), Klosterneuburg, Austria; 5 Laboratory of Molecular Genetics, N.F. Gamaleya National Research Center for Epidemiology and Microbiology, Ministry of Health of the Russian Federation, 123098 Moscow, Russia; 6 Laboratory of Immunogenetics, Institute of Molecular and Cellular Biology, Siberian Branch of the Russian Academy of Sciences, 630090 Novosibirsk, Russia

**Keywords:** Vaccines, Immunological memory

## Abstract

Replication-incompetent adenoviral vectors have been extensively used as a platform for vaccine design, with at least four anti-COVID-19 vaccines authorized to date. These vaccines elicit neutralizing antibody responses directed against SARS-CoV-2 Spike protein and confer significant level of protection against SARS-CoV-2 infection. Immunization with adenovirus-vectored vaccines is known to be accompanied by the production of anti-vector antibodies, which may translate into reduced efficacy of booster or repeated rounds of revaccination. Here, we used blood samples from patients who received an adenovirus-based Gam-COVID-Vac vaccine to address the question of whether anti-vector antibodies may influence the magnitude of SARS-CoV-2-specific humoral response after booster vaccination. We observed that rAd26-based prime vaccination with Gam-COVID-Vac induced the development of Ad26-neutralizing antibodies, which persisted in circulation for at least 9 months. Our analysis further indicates that high pre-boost Ad26 neutralizing antibody titers do not appear to affect the humoral immunogenicity of the Gam-COVID-Vac boost. The titers of anti-SARS-CoV-2 RBD IgGs and antibodies, which neutralized both the wild type and the circulating variants of concern of SARS-CoV-2 such as Delta and Omicron, were independent of the pre-boost levels of Ad26-neutralizing antibodies. Thus, our results support the development of repeated immunization schedule with adenovirus-based COVID-19 vaccines.

## Introduction

Replication-incompetent, recombinant adenovirus (rAd) vectors are an excellent platform for producing vaccines against various human pathogens^1^. For example, development of rAd-based vaccines was reported for human immunodeficiency virus type 1 (HIV-1)^2^, Ebola^3^, Zika^4^, and some others viruses, and this platform has become particularly popular during the on-going COVID-19 pandemic caused by SARS-CoV-2. Currently, the most advanced rAd-based COVID vaccines include Ad26.COV2.S (Jcovden, Janssen), Ad5-nCoV (Convidecia, CanSino), AZD1222 (Vaxzevria, AstraZeneca), and Gam-COVID-Vac (Sputnik V, Gamaleya Institute)^5–8^.

Vaccination has proven to be highly effective in reducing the risk of symptomatic disease, morbidity, and mortality rate in SARS-CoV-2-infected individuals^9^. However, vaccine-induced immunity is known to wane with time, and in order to maintain the protective levels of cellular and humoral immunity, booster vaccination(s) are highly warranted^10^. Also, the need for revaccination is substantiated by the emergence of novel viral variants of concern (VOCs), which have been shown to evade neutralization by the antibodies formed after infection or vaccination. Revaccination helps overcome this immune evasion, as administration of a booster dose has led to a broader and more durable immune protection^11–13^. Yet, when multiple rounds of vaccination and revaccination are envisaged, this is inevitably accompanied by immune responses to vaccine components unrelated to the immunogen, and results in mounting anti-vector immunity^9^. One of the numerous advantages of mRNA-based vaccines is that anti-carrier immune responses they produce are extremely rare^14^, hence boosting does not appear to be problematic for this platform. In contrast, rAd-based vaccines are known to result in anti-vector immunity, and the dynamics and magnitude of this response is of significant concern. Negative effects of anti-vector immunity on revaccination success were demonstrated previously^15–17^. In the mouse model, the half-life of anti-Ad5 neutralization antibodies (NAbs) was approximately 6 months^18^. Further, pre-existing anti-Ad5 immunity was shown to suppress the immunogenicity of rAd5-vectored vaccines in animals and humans^19,20^.

One distinctive feature of Gam-COVID-Vac is that it is a two-component vaccine, i.e., it is based on two adenoviral vectors. In a standard regimen, the first and the second vaccine doses are delivered as non-replicating recombinant human adenovirus type 26 (rAd26) and type 5 (rAd5) particles, respectively^8^. Both vectors carry the cassette encoding unmodified ancestral (WT) full-length SARS-CoV-2 Spike glycoprotein. Switching between two serologically distinct viral vectors aims to address the issue of vaccine neutralization by anti-vector antibodies, however, upon boosting and/or subsequent repeated rounds of immunization with the same platform, this may again become problematic.

Here, we asked whether the humoral anti-SARS-CoV-2 immunity after Gam-COVID-Vac revaccination depends on the level of anti-vector post-prime antibodies. Also, we aimed to describe the dynamics and longevity of anti-Ad26 NAb responses over the period of 14 months. We show that both RBD-binding and SARS-CoV-2 NAb recall responses are independent of the pre-boost levels of Ad26 NAbs. Our findings therefore help determine the optimal timing of the booster dose(s) for rAd-based vaccines.

## Results

### Booster Gam-COVID-Vac vaccination elicits high titers of serum Ad26-neutralizing antibodies

**Fig. 1:**
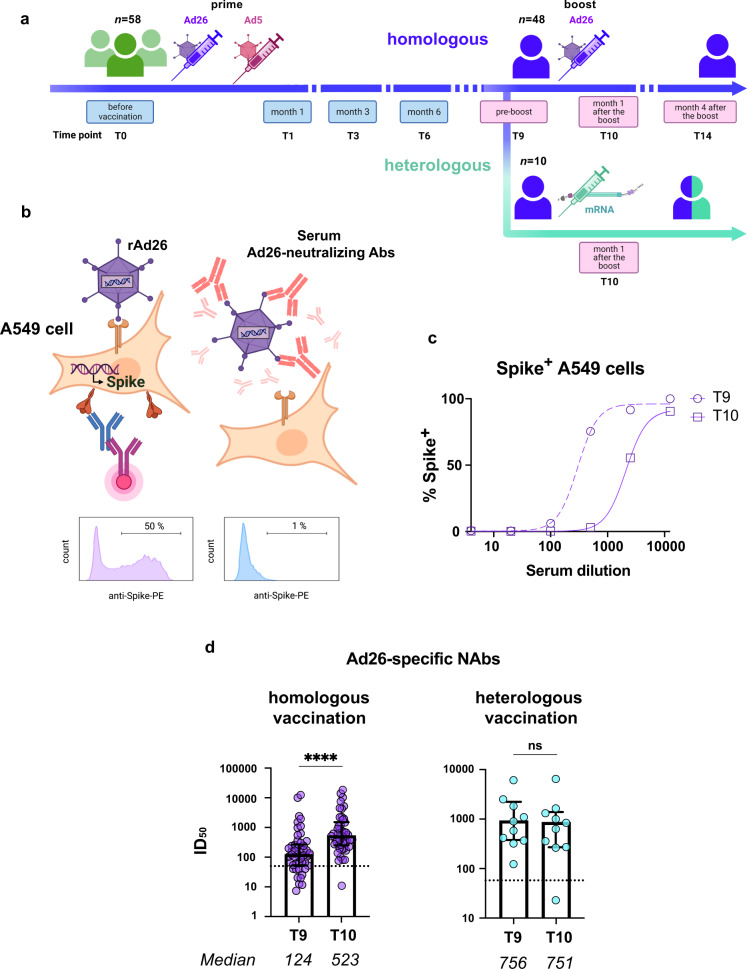
Booster vaccination with rAd26-vectored Gam-COVID-Vac elicits high titers of serum Ad26-specific NAbs. **a** Timeline showing Gam-COVID-Vac vaccination and blood sampling timepoints, labeled according to the number of months elapsed after the first dose. **b** Outline of the rAd26 virus neutralization assay. In a subpanel, representative flow plots show Spike expression on the surface of A549 cells after rAd26-Spike infection in the absence of Ad26-neutralizing antibodies (left) and in the presence of a saturating amount of Ad26 Nab (right). Any anti-Spike activity in serum samples was blocked by adding the excess of recombinant Spike protein (not shown). **c** Representative rAd26 neutralization curves for serum samples before (T9) and one month after (T10) the Gam-COVID-Vac boost. Ad26 neutralizing activity was defined by the percentages of Spike^+^ A549 cells relative to no antibody controls. **d** Serum Ad26 virus-neutralizing antibody titers before and one month after booster vaccination in homologous (Gam-COVID-Vac/Gam-COVID-Vac) and heterologous (Gam-COVID-Vac/Pfizer-BioNTech’s Comirnaty) vaccination groups. Dotted lines indicate the pre-prime baseline. Medians are plotted and statistical significance was determined using Wilcoxon matched-pairs signed-rank test. Data are presented as median values and interquartile ranges (IQR). ****P < 0.0001.

Fifty-eight individuals were recruited to our study. All study participants were vaccinated with two injections of Gam-COVID-Vac in January–April, 2021. The first dose was rAd26-based, followed by the rAd5-based second dose 21 days later. After 9 months [range 249–300 days, Supplementary Table S1], all participants received their booster vaccination. In the core cohort (*n* = 48), rAd26-based first component of the Gam-COVID-Vac served as a booster, and in the comparison cohort (*n* = 10), the booster was Pfizer-BioNTech’s Comirnaty. Serum samples were collected at baseline (T0), one, three and six months later (T1, T3, and T6), and then immediately before, and one and four months after the booster immunization (T9, T10, and T14, respectively) (Fig. 1a).

The outline of the rAd26-neutralization assay used in our study is presented in Fig. 1b. A549 human lung carcinoma cells were used as the target for rAd26 infection. After 24 h of infection, approximately 50% of A549 cells expressed the full-length SARS-CoV-2 glycoprotein S encoded by the rAd26 vector, which was detected using anti-RBD monoclonal antibody XR15 (Fig. 1b, left panel). In the presence of saturating amount of anti-Ad26 NAbs, the infection was blocked, and no S expression was detectable (Fig. 1b, right panel). Sera obtained from the vaccinated individuals inhibited rAd26 vector entry in a concentration-dependent manner (Fig. 1c) and the level of anti-Ad26 NAbs was quantified as neutralization half-maximal inhibitory serum dilution (ID_50_). Usually, Ad NAb titers are assessed using rAd-luciferase or rAd-GFP reporter constructs^21,22^. However, we believe that the use of rAd directly from the Gam-COVID-Vac vaccine preparation provides more relevant information that is closer to the real-life situation.

First of all, we measured the titers of anti-vector antibodies before (T9) and after (T10) the booster Gam-COVID-Vac vaccination, in a core cohort with a homologous regimen of revaccination (i.e., in study participants who received Gam-COVID-Vac as both the prime and the boost). Sera from the vaccinated individuals sampled at a pre-boost timepoint T9 were found to noticeably inhibit infection of A549 cells with rAd26 vector (median = 124, Fig. 1d), with 79% (38/48) of the samples displaying anti-Ad26 NAb levels above the baseline (ID_50_ for healthy non-vaccinated donors, median = 50). These numbers are consistent with the fact that all study participants had their first dose of Gam-COVID-Vac, which is rAd26-based, 9 months earlier. Following revaccination, Ad26-NAb titers increased 4.2-fold (median ID_50_ = 523, *P* < 0.0001), compared to the pre-boost levels, and the anti-Ad26 seroprevalence reached 98%.

Individuals from the comparison arm received a heterologous boost (i.e., different platform/delivery vector/Spike). All participants who received an mRNA vaccine booster *(n* = 10), had unaltered Ad26 NAb levels (*P* = 0.3223). The levels of Ad5-specific NAbs remained the same in both homologous and heterologous vaccination groups (*P* = 0.0608; *P* = 0.1602, correspondingly) (Supplementary Fig. S1).

Thus, rAd26-based but not mRNA-based revaccination predictably led to the formation of anti-vector responses in all of the vaccinees. This is consistent with the idea that the increase in Ad26-neutralizing titers is due to the administration of rAd26-based vector, rather than the vaccination per se.

### RBD-binding and SARS-CoV-2-neutralizing antibody titers are independent of the level of pre-boost anti-Ad26 antibodies

**Fig. 2:**
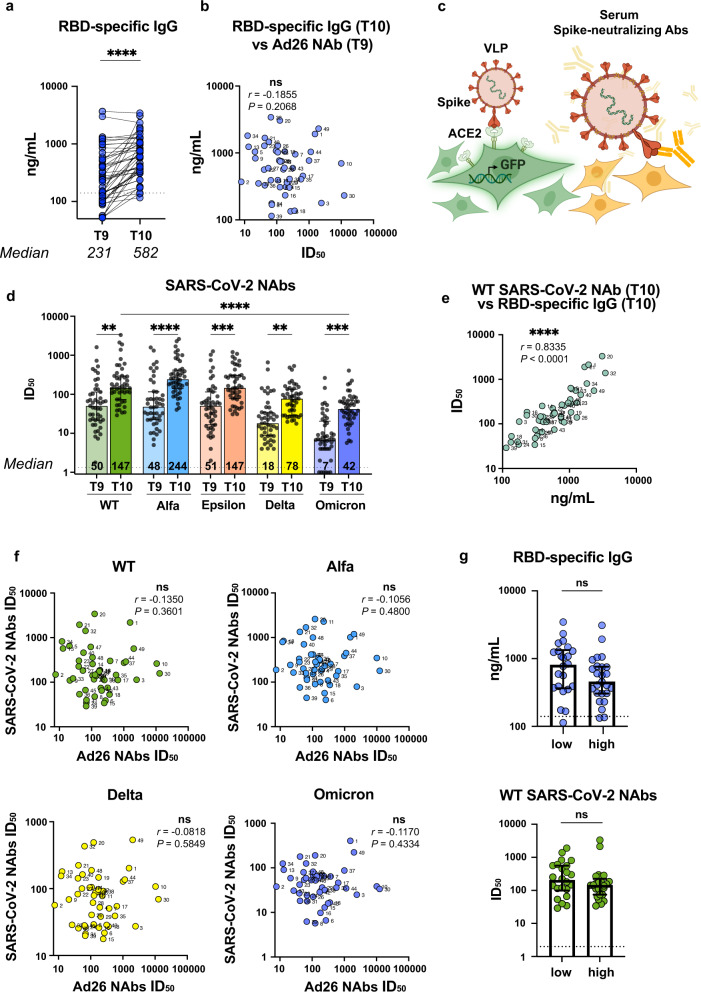
Humoral anti-SARS-CoV-2 recall responses after booster Gam-COVID-Vac vaccination. **a** Serum anti-RBD IgG levels before and one month after the booster injection measured by ELISA. **b** Spearman’s correlation between pre-boost (T9) Ad26-specific NAb values (*x* axis) and the serum levels of anti-RBD IgGs at T10 (y axis). Participant IDs are shown. **c** Schematic representation of the SARS-CoV-2 virus neutralization assay. **d** Neutralizing antibody titers (ID_50_) against SARS-CoV-2 WT, Alfa, Epsilon, Delta, and Omicron VOCs. Numbers indicate median values. **e** Spearman’s correlation between RBD-specific IgG and WT Spike SARS-CoV-2 NAbs at T10. **f** Lack of significant association between pre-boost (T9) Ad26-specific NAb levels with post-boost (T10) SARS-CoV-2 NAb titers against WT, Alfa, Epsilon, Delta, or Omicron VOCs. Numbers indicate Spearman’s rank correlation coefficients. **g** Comparison of RBD-binding IgG levels and SARS-CoV-2 Nab titers at T10 in subgroups with low (below the median) and high (above the median) Ad26 NAb ID_50_ at pre-boost timepoint T9. Medians are plotted and statistical significance was determined using Wilcoxon matched-pairs signed-rank test (**a**), Kruskal–Wallis test with Dunn’s post correction (**d**) or Mann–Whitney test (**g**).

Of all Spike-specific antibodies, RBD-targeting antibodies are known to constitute the vast majority of SARS-CoV-2 NAbs^2^, hence in order to measure recall humoral responses after booster vaccination we first analyzed the levels of anti-RBD IgGs. Nine months after the prime and immediately before the booster shot (timepoint T9), study participants displayed pronounced RBD-specific IgG titers (median = 231 ng/mL). Seroprevalence of anti-RBD IgG was 60% (29/48). As expected, Gam-COVID-Vac revaccination resulted in a 2.5-fold increase in RBD-specific IgG levels (median = 582 ng/mL, *P* < 0.0001) and seroprevalence 94% (45/48) (Fig. 2a). An even more pronounced increase in RBD-specific IgGs was observed in the heterologous vaccination group (8.7-fold change, *P* = 0.0039) (Supplementary Fig. S2).

To understand whether the level of pre-boost Ad26-specific immunity may affect the levels of post-boost Spike-specific antibodies, we tested for associations between the levels of RBD-specific IgGs at T10 vs. Ad26-specific NAbs at T9 (Fig. 2b). The Spearman’s correlation analysis did not reveal any association (Spearman *r* = −0.1855, *P* = 0.2068), indicating that pre-boost anti-Ad26 NAbs did not affect the production of RBD-binding IgGs.

While RBD-binding activity of antibodies is known to be important for protective immunity against SARS-CoV-2, their functional activity, i.e., the ability to neutralize the virus, may serve as a better predictor of protection. To quantify the SARS-CoV-2-neutralizing activity of sera from vaccinated individuals, virus neutralization assay was performed using lentiviral particles pseudotyped with Spike from SARS-CoV-2 WT (ancestral variant) or VOCs (Fig. 2c).

Before the booster at T9, serum samples demonstrated ID_50_ ~50 for WT, Alfa, and Epsilon VOCs of SARS-CoV-2. In addition, these sera also neutralized Delta and Omicron variant entry, albeit the latter was neutralized less effectively compared to the WT (*P* < 0.0001) (Fig. 2d).

Much as was observed for RBD-specific IgG responses, the WT SARS-CoV-2 neutralizing capacity of the sera significantly increased after the booster shot (T10) compared with the pre-booster (T9) levels (*P* = 0.0017). Furthermore, this was accompanied by higher neutralizing activities against Alfa, Epsilon, Delta, and Omicron VOCs (fold increase 4.0, 2.4, 2.3, and 3.9, respectively) (Supplementary Fig. S3). In the heterologous vaccination group receiving an mRNA-based boost, anti-SARS-CoV-2 NAb levels against WT, Alfa, Epsilon, Delta, and Omicron were even higher (20.9, 31.6, 19.1, 15.2, and 47.0-fold increase, respectively) (Supplementary Fig. S4).

We next sought for possible associations between the immunological parameters assayed. As expected, a good correlation between RBD-specific IgGs and WT Spike SARS-CoV-2 NAbs at T10 was observed (Spearman *r* = 0.8335, *P* < 0.0001) (Fig. 2e). At the same time, we failed to detect any association between the levels of Ad26-specific NAbs at T9 and SARS-CoV-2-neutralizing activities against WT or other tested VOCs (Alfa, Epsilon, Delta, and Omicron) (Spearman *r* = −0.1350, *P* = 0.3601; *r* = −0.1056, *P* = 0.4800; *r* = −0.0356, *P* = 0.8121; *r* = −0.0818, *P* = 0.5849; *r* = −0.1170, *P* = 0.4334, respectively, Fig. 2f, Supplementary Fig. S5). Next, the vaccinated individuals were stratified as having their Ad26 NAb ID_50_ values at the pre-boost timepoint T9 below or above the median. After booster vaccination (T10), the stratified subgroups did not differ in the levels of either RBD-binding IgG or SARS-CoV-2 NAb (*P* = 0.0948 and *P* = 0.1954, respectively, Fig. 2g). Taken together with the correlation analysis, these data suggest that both RBD-binding and SARS-CoV-2 NAb recall responses are independent of the pre-boost levels of Ad26-specific NAbs.

### Dynamics of Ad26 virus-neutralizing, SARS-CoV-2 RBD-binding antibodies, and SARS-CoV-2 NAbs

**Fig. 3:**
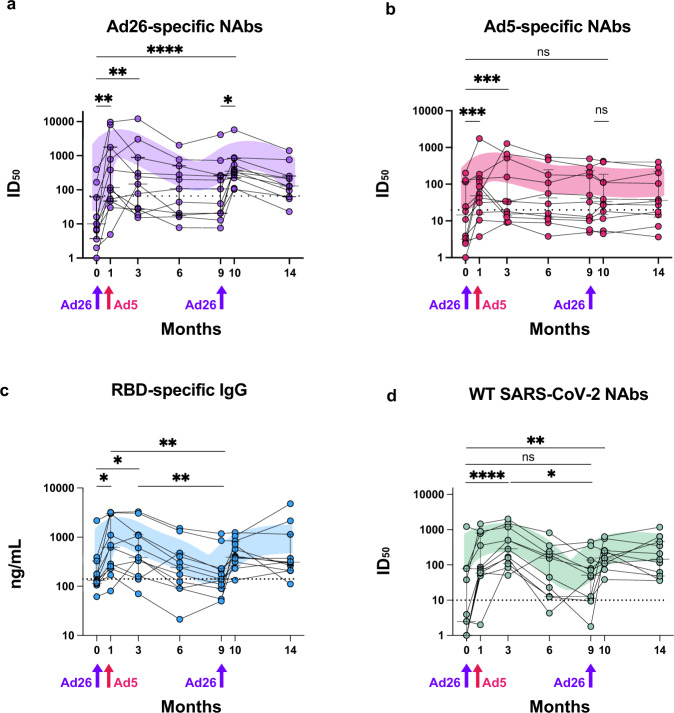
Longitudinal analysis of humoral responses to Gam-COVID-Vac vaccination. Ad26-specific NAb (**a**), Ad5-specific NAb (**b**), anti-RBD IgG (**c**), and WT SARS-CoV-2 NAb (**d**) responses before the first dose of Gam-COVID-Vac and up to 14 months later. LOWESS-smoothed lines are shown. Arrows indicate immunization timepoints. Asterisks indicate significant difference between groups determined using the Friedman test.

Our analysis described above has established the increase in anti-Ad26 antibody titers following homologous revaccination. We asked when these antibodies appeared during the course of vaccinations and how long would these antibodies persist at detectable levels. In a subcohort of donors (*n* = 11), Ad26-specific NAb levels were monitored longitudinally from the first dose of the vaccine and up to 14 months at 7 timepoints. Figure 3a illustrates the dynamics of Ad26 neutralizing antibodies across six time intervals.

Pre-vaccine median level of Ad26 NAbs was on the baseline. It must be noted though that two individuals in our cohort had high pre-existing Ad26 NAb titers. One month after the prime vaccination, the levels of Ad26 NAbs demonstrated a marked increase, and slightly declined thereafter, yet persisted for the duration of 6 months of the follow-up. During the study, Ad26 NAb seroprevalence was invariably above 46%. Booster immunization restored Ad26 NAb levels back to the maximum levels, which was again followed by a minor decline. The magnitudes of Ad26 NAb peaks following prime and booster vaccination were generally comparable, and a 14.7- and 34.8-fold increase was observed relatively to the baseline (*P* = 0.0025, *P* < 0.0001, respectively). In contrast, the Ad5 response dynamics had a single peak soon after the prime Ad5-based vaccination (T1 vs. T0, 5.0-fold, *P* = 0.0007) and then gradually decreased to baseline at T14 (Fig. 3b).

The dynamics of RBD-specific IgG and WT SARS-CoV-2 NAb responses followed the pattern observed for Ad26 NAbs (Fig. 3c, d). Namely, after the second shot, RBD-binding IgG and WT SARS-CoV-2 NAb levels progressively declined for 6 months and reached a minimum before revaccination. Notably, RBD-binding antibody levels demonstrated the strongest decline, with nearly background levels found by the 9th month of the study. Nonetheless, WT SARS-CoV-2 NAb activity remained clearly detectable. Based on these numbers, setting a booster shot at 6–9 months after the prime appears well-substantiated, as this offers the advantage of declining Ad26 NAb titers with persisting SARS-CoV-2 humoral immunity.

## Discussion

Gam-COVID-Vac was originally designed as a two-dose vaccine minimizing the production of anti-vector antibodies. Ad26 serotype-based vector was purposefully selected as the first dose component. Due to its known low prevalence in the human population, pre-existing anti-Ad26 antibodies would be expected to be less of a problem. The second Gam-COVID-Vac component is based on the rAd5 vector, which means that the vaccine remains homologous in terms of the SARS-CoV-2 Spike expression, yet is heterologous vector-wise and may evade the elicited Ad26-specific NAb responses. Despite the fact that Ad5 seroprevalence is significantly higher than that of Ad26, pre-existing Ad5 NAbs do not seem to present a substantial issue upon the second vaccine dose^8^.

Real-life experience with Gam-COVID-Vac and other vaccines has indicated that efficient immunization against SARS-CoV-2 requires triple or even multiple rounds of vaccine administration, particularly after the emergence of Delta and Omicron viral lineages that are poorly neutralized by one or two rounds of the WT Spike-based vaccines^11–13^.

In the current study, we observed a robust anti-Ad26 NAb response after the prime vaccination with Gam-COVID-Vac, which agrees well with the recent report^22^. One month after the first injection of the rAd26-based vaccine component, Ad26-neutralizing reciprocal geometric mean titer (GMT) levels increased at least 40-fold. Having compared the levels of anti-rAd26 viral vector NAbs before the vaccination and the titers of RBD-specific IgGs one month later, baseline anti-Ad26 NAb levels were concluded not to affect the anti-SARS-CoV-2 immunity elicited upon a single-dose Gam-COVID-Vac vaccination.

Importantly, our study goes one step further in that we assessed the possible interplay between anti-SARS-CoV-2 responses and pre-boost vector-targeted anti-Ad26 antibodies 9 months after the prime vaccination. Despite the fact that pre-boost Ad26 NAb levels increased ~11-fold compared to the pre-vaccination levels, this had no influence on the downstream anti-SARS-CoV-2 humoral response. It is therefore plausible to assume that if boosting is scheduled when Ad26-NAb titers are the highest, this may not be as neutral and have negative consequences for the mounting anti-SARS-CoV-2 response.

Our data are also in excellent agreement with previous studies demonstrating that a second or subsequent doses of Ad26-based Ad26.EnvA vaccine induced humoral responses to HIV antigens even in the presence of high vector-induced Ad26 NAb titers^2^.

Anti-vector humoral immunity is also a well-appreciated issue for other Ad-based vaccines^23^. Currently available Ad-vectored COVID-19 vaccines provide genetic information for the biosynthesis of largely the same Spike protein, but differ in the choice of the vector serotypes.

Ad26.COV2.S developed by Janssen Pharmaceuticals was originally designed as a single-shot vaccine, however, a two-dose regimen was later chosen to increase protection^24^. As Ad26.COV2.S is Ad26-based, our results are to a certain extent applicable to the possible use of this vaccine in a prime-boost regimen. Given that ChAdOx1 nCoV-19 vaccine produced by The University of Oxford-AstraZeneca under the brand name Vaxzevria is based on a single non-replicating chimpanzee adenovirus Y25, anti-vector antibodies may be elicited and therefore interfere with the immunization in a standard two-dose regimen. Three-dose vaccination with ChAdOx1 nCoV-19 has recently been reported^25^, which makes the issue of anti-vector immunity even more relevant. Therefore, our results on the apparent lack association between the pre-boost anti-Ad26 antibody responses and the post-boost SARS-CoV-2 NAb titers may likely be extrapolated to the second and the third doses of ChAdOx1 nCoV-19.

Finally, it must be kept in mind that prime-induced anti-vector immunity has a T cell component, which may influence the outcome of subsequent Ad-based vaccination steps^26^ and should be explored separately.

To summarize, we report here that pre-existing anti-vector antibodies do not appear to compromise the efficacy of the booster Gam-COVID-Vac vaccination, as assayed by anti-RBD and SARS-CoV-2 NAb titers. The most parsimonious explanation of our data is that the vector particles injected in a vaccine simply outnumber the neutralizing antibodies, so their possible negative influence would be negligible. Yet, it should be taken into account that anti-vector antibodies may not only suppress, but also stimulate immune response via the mechanism of Fc-receptor mediated viral entry. This mechanism is central to the phenomenon referred to as antibody-dependent enhancement (ADE) of infection, which has been described for a number of vaccines developed against respiratory syncytial virus, influenza, and dengue, as well as for the cases of secondary infections^27–29^. Binding of sub-neutralizing antibodies can enhance viral fusion mediated by Fc-receptors that are expressed on immune cells, which ultimately translates into activation of B and T cells. Thus, the observed lack of influence of pre-existing anti-vector antibodies on the NAb titers may well result from the mutual compensation of the two opposing effects of ADE and antibody-dependent neutralization of adenovirus particles.

In conclusion, to our knowledge, this is the first report of the possible impact of prime-induced anti-vector neutralizing antibodies on the efficacy of Ad-vectored COVID-19 vaccine. Our observation that anti-vector antibodies do not appear to affect the anti-SARS-CoV-2 immunity is quite encouraging, as it supports performing multiple immunizations with adenovirus-based COVID-19 vaccines.

## Methods

### Volunteers and ethics

A cohort of 58 volunteers was enrolled at the National Research Center Institute of Immunology of The Federal Medical Biological Agency of Russia. Between January and April 2021, all subjects received two doses of Gam-COVID-Vac vaccine. The first dose was rAd26-based, followed by the rAd5-based second dose 21 days later. Approximately 9 months after the prime, the booster vaccine dose was given. The core cohort participants received a homologous Gam-COVID-Vac (rAd26) boost, whereas the comparison cohort had a heterologous boost with the Pfizer-BioNTech’s Comirnaty.

Written informed consent was obtained from each of the study participants before performing any study procedures. The study protocol was reviewed and approved by the Medical Ethical Committee of Institute of Immunology (#12-1, December 29, 2020).

### Elisa

The level of SARS-CoV-2 receptor binding domain (RBD)-specific antibodies was measured using ELISA Quantitation Kit (Xema Co.)^30^. Briefly, plasma samples were 5-fold serially diluted from 1:20 to 1:12500 in blocking buffer. Plates were incubated with samples for 1 h at room temperature. The plate was washed thrice, followed by the addition of a 1:5000 dilution of anti-human IgG conjugated to horseradish peroxidase (Jackson Immuno Research, Cat# 009-030-008) and incubation for 1 h. ELISA plates were washed 7 times and developed for 10 min with 100 μL of tetramethylbenzidine chromogen solution. The reaction was stopped with 50 μl 1 M H_2_SO_4_ and absorbance at 450 nm was read with an iMark microplate absorbance reader (Bio-Rad). Each sample was measured in triplicate. To determine the concentration of IgGs, a serial dilution of anti-SARS-CoV-2 RBD-specific human monoclonal antibody iB12^31^ was included on each plate, a calibration curve was built and IgG levels were calculated (μg/mL).

### Recombinant Ad26 and Ad5 neutralization assay

First of all, any anti-Spike activity in serum samples was blocked by adding recombinant Spike protein (160 µg/mL) and incubation for 3 h. Blocking efficiency for each sample was confirmed by ELISA using Spike coated plates (Supplementary Fig. S6). Five-fold serial dilutions of blocked serum samples in cell culture medium were made and combined with an equal volume of rAd and incubated for additional 1 h. The first and the second dose of the Gam-COVID-Vac vaccine were used as the source of recombinant Ad26 or Ad5 vector particles, respectively. After incubation, serum/rAd/Spike mixture (40 μL) was mixed with an equal volume of 50,000 target cells (A549), and seeded into wells of 96-well plates. After incubation for 24 h in a standard CO_2_ incubator (37 °C, 5% CO_2_), cells were harvested, washed and Ad-mediated SARS-CoV-2 Spike expression on the surface of infected A549 cells was quantified using indirect surface staining. Cells were first incubated with an anti-WT RBD SARS-CoV-2 mouse antibody (clone XR15 was a gift from Dr. Yuri Lebedin) at 5 μg/mL for 30 min at room temperature to identify infected cells. Antibody binding was then detected using goat anti-mouse IgG-PE (BioLegend, Cat# 405307) (2.5 μg/mL, 30 min at room temperature in the dark). Finally, immunofluorescence signal was quantified using CytoFLEX S flow cytometer. Cells stained with isotype-matched irrelevant mouse monoclonal antibody served as a negative control. Data were analyzed using FlowJo Software (version 10.6.1., Tree Star). Preparations of Ad26 and Ad5 were preliminarily titrated to infect 50% of target cells after 24 h of infection, which corresponded to ~2 × 10^8^ viral particles. rAd neutralizing activity was measured at six dilution levels: 4, 20, 100, 500, 2500, and 12,500 for each sample. Neutralization titers (ID_50_) were calculated using GraphPad Prism 8 and defined as the reciprocal serum dilution at which the % of Spike^+^ A549 cells was reduced by 50% compared to no-rAd control wells after subtraction of background signal in the negative control. The baseline level of anti-rAd NAb was determined using serum samples of healthy non-vaccinated donors (*n* = 16), which were not included in the study cohort.

### SARS-CoV-2 pseudovirus production

To produce SARS-CoV-2 Spike-pseudotyped lentiviral particles, 3 plasmids were used: HIV-1 packaging pCMVΔ8.2R (Addgene), transfer pUCHR-GFP (Addgene), and envelope pCAGGS-Swt- Δ19^32^. The latter plasmid encodes a codon-optimized ancestral Wuhan-Hu-1 Spike(Δ19) lacking 19 C-terminal amino acid residues. Based on pCAGGS-Swt-Δ19, several plasmids encoding SARS-CoV-2 Spike variants were obtained by site-specific mutagenesis or gene synthesis (Genewiz). Specifically, these Spike variants had the following substitutions: delta69-70, delta144, N501Y, A570D, D614G, P681H, T716L, S982A, D1118A (Alfa); S13I, W152C, L452R, D614G (Epsilon); T19R, T95I, 156del, 157del, R158G, L452R, T478K, D614G, P681R, D950N (Delta); A67V, Δ69–70, T95I, G142D, Δ143–145, Δ211, L212I, ins214EPE, G339D, S371L, S373P, S375F, K417N, N440K, G446S, S477N, T478K, E484A, Q493R, G496S, Q498R, N501Y, Y505H, T547K, D614G, H655Y, N679K, P681H, N764K, D796Y, N856K, Q954H, N969K, L981F (Omicron BA.1).

Lentiviral particles pseudotyped with the SARS-CoV-2 S protein of WT strain or VOCs were produced as described^32^. The viral particles were immediately used in neutralization tests or stored at −70 °C. Viral yield was quantified using titration on HEK293-hACE2 cells.

### Pseudotyped SARS-CoV-2 neutralization assay

SARS-CoV-2 pseudovirus neutralization assay was performed as described previously^32^. Briefly, the 293T-ACE2 cells (2 × 10^4^ cells/well) were seeded in 96-well plates. For the neutralization assay, pseudovirus suspension (20 µL) was mixed with an equal volume of serial serum dilutions (1:20, 40, 80, 160, 320, 640, 1280, 2560, 5120, 10,240, and 20,480) and incubated for 1 h at 37 °C. The mixture was then added to 50 µL of 293T-hACE2 cells in a 96-well plate and incubated for two days (37 °C, 5% CO_2_). The RBD-specific neutralizing human monoclonal antibody iB12^31^ was used as a positive control. ID_50_ was determined by non-linear regression.

### Statistical analysis

Statistical significance was determined using Wilcoxon matched-pairs signed-rank test, Kruskal–Wallis test with Dunn’s post correction, Friedman or Mann–Whitney test. Non-parametric Spearman correlations were used to determine associations between the analyzed immunological parameters. Statistical analysis was performed using Graph Pad Prism (version 9.3.1 GraphPad Software). ID_50_ values for Ad and SARS-CoV-2 neutralization were analyzed by non-linear regression to estimate the reciprocal dilution of sera required for half-maximal neutralization of infection (ID_50_ titer) and were calculated using GraphPad Prism software (Sigmoidal, 4PL). *P* < 0.05 was considered statistically significant. Data are presented as median ± IQR. Non-parametric LOWESS (Locally Weighted Scatterplot Smoothing) was used for smoothing. Asterisks indicate significant difference between groups, *P < 0.05, **P < 0.01, ***P < 0.001, ****P < 0.0001, ns = not significant.

### Reporting summary

Further information on research design is available in the Nature Research Reporting Summary linked to this article.

## Supplementary information

Supplemental material

REPORTING SUMMARY

## Data Availability

The raw de-identified data that support the findings of this study are available from the corresponding author upon reasonable request.

## Code availability

No custom code or mathematical algorithm was used for the analysis of the data.
