# Supplementary material for: Anti-Ad26 humoral immunity does not compromise SARS-COV-2 neutralizing antibody responses following Gam-COVID-Vac booster vaccination
Source: NPJ Vaccines. 2022 Nov 15;7:145. doi: 10.1038/s41541-022-00566-x (PMC9665038; doi:10.1038/s41541-022-00566-x)
Supplement: Supplementary file 1 — Supplemental material [file 41541_2022_566_MOESM1_ESM.pdf]

Supplementary Table S1. Cohort description.

**Regimen of vaccination**	**Homologous**	**Heterologous**
**Number of participants**	48	10
**Age,**<br>years, median (range)	27 (18 – 73)	27.5 (23 – 39)
Female<br>Male	23<br>25	4<br>6
**First dose**<br>Gam-COVID-Vac (Ad26)	48	10
**Second dose**<br>Gam-COVID-Vac (Ad5)	48	10
**Booster vaccine**<br>Gam-COVID-Vac (Ad26)<br>Pfizer	48<br>0	0<br>10
**Time between prime vaccination and booster**<br>days, median (range)	273 (249 – 300)	282 (267 – 328)
**Probe sampling after booster,**<br>days, median (range)	33 (29 – 41)	42 (38 – 49)
Fever<br>Injection site reactions<br>Weakness<br>Headache	14 (29%)<br>22 (46%)<br>21 (44%)<br>6 (13%)	6 (60%)<br>9 (90%)<br>7 (7%)<br>2 (2%)

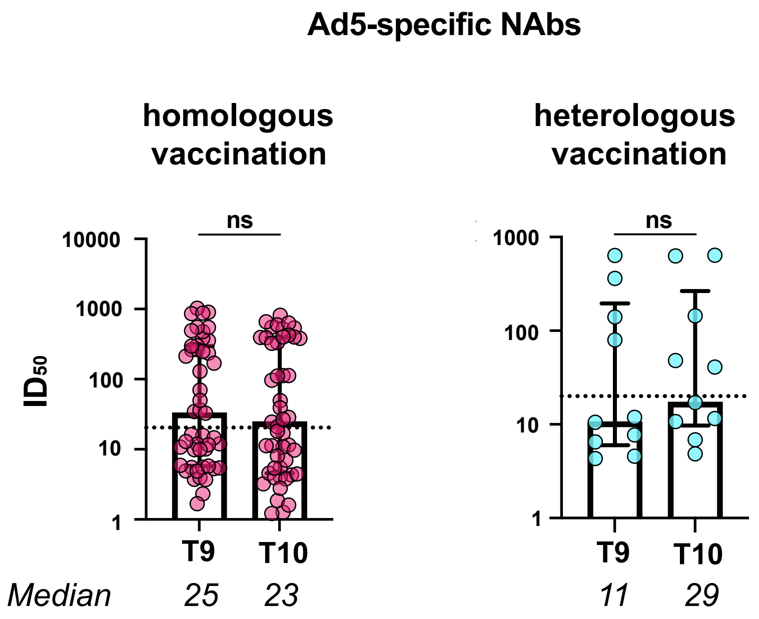

Supplementary Figure S1. The levels of Ad5-specific NAbs before (T9) and after (T10) homologous (Gam-COVID-Vac/Gam-COVID-Vac) or heterologous (Gam-COVID-Vac/mRNA) vaccination boost.

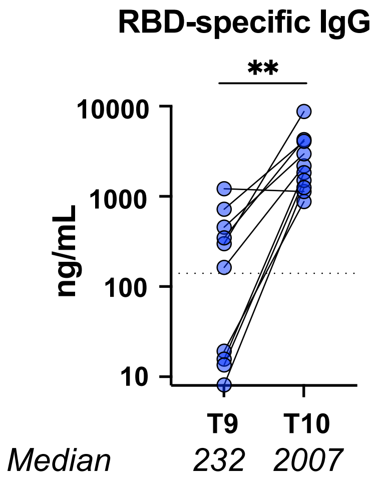

Supplementary Figure S2. RBD-specific IgG response in the heterologous vaccination group before (T9) and after (T10) boost mRNA vaccination.

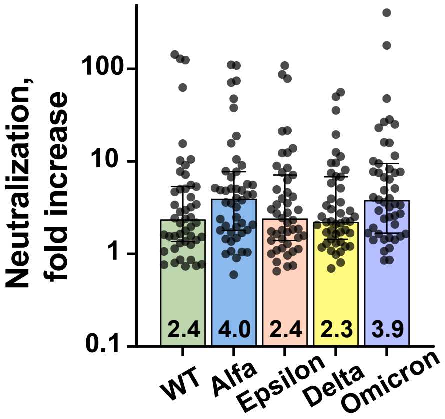

Supplementary Figure S3. Fold increase in SARS-CoV-2 neutralizing activity of sera after boost Gam-COVID-Vac vaccination. Virus neutralization data presented for SARS-CoV-2 wild type (WT), Alfa, Epsilon, Delta, and Omicron mutant variants. Numbers inside bars indicate median fold increase.

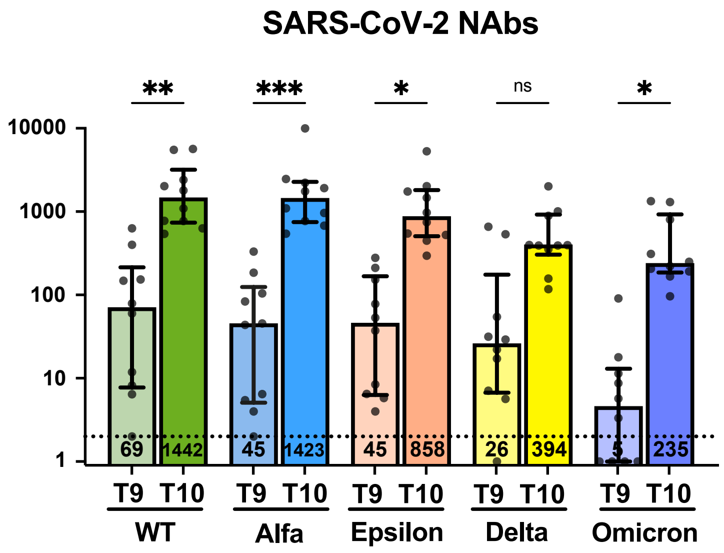

Supplementary Figure S4. SARS-CoV-2 NAbs responses in the heterologous vaccination group against WT, Alfa, Epsilon, Delta, and Omicron VOCs after boost mRNA vaccination. Median values are indicated.

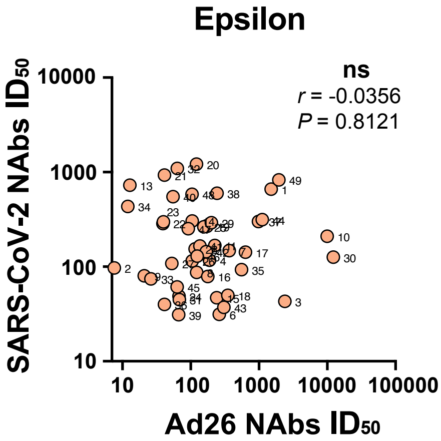

Supplementary Figure S5. Spearman's correlation between pre-boost (T9) Ad26-specific NAb levels and post-boost (T10) SARS-CoV-2 NAbs titers against Epsilon VOC.

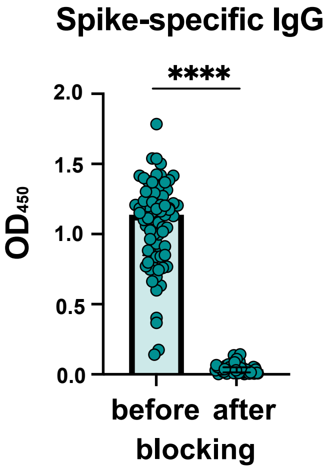

Supplementary Figure S6. ELISA testing of anti-Spike activity in serum samples before and after blocking with recombinant Spike protein (160 µg/mL).
